# Profiling nursing students’ dishonest behaviour: Classroom versus clinical settings

**DOI:** 10.1177/09697330221075779

**Published:** 2022-05-26

**Authors:** Robert Lovrić, Boštjan Žvanut

**Affiliations:** 84992 Faculty of Dental Medicine and Health Osijek, Josip Juraj Strossmayer University of Osijek, Croatia; 68960 Faculty of Health Sciences, University of Primorska, Slovenia

**Keywords:** Ethics education, academic dishonesty, clinical settings, classroom, dishonest behaviour

## Abstract

**Background:**

While academic dishonesty among nursing students is becoming a global problem, the instruments used in studies on this topic are heterogeneous and, in many cases, not even validated. This makes it difficult or impossible to compare the findings on a global scale.

**Objectives:**

To investigate the profile of Croatian nursing students’ dishonest behaviour in classroom and clinical settings and to examine the relationship between the incidence of dishonest behaviour in both settings.

**Research design:**

A quantitative cross-sectional study using a Croatian online version of the *Nursing Student Perceptions of Dishonesty Scale* (overall Cronbach’s alpha 0.933)*.*

**Participants and research context:**

446 nursing students from a higher education institution in Croatia, EU, in the academic year 2020/21.

**Ethical considerations:**

The study was approved by the relevant committee of the higher education institution.

**Findings/results:**

Almost all participants (91.3%) performed dishonest behaviour on two or more occasions in the classroom and 32.5% did so in the clinical setting. The incidence of dishonest behaviour increased with the students’ year of BSc study (*p* = .008). All subscales of dishonest behaviour in the classroom were significantly and positively associated with dishonest behaviour in the clinical setting, except for the *Not My Problem* and *Non-Compliance* subscales.

**Discussion:**

Based on these results, the following should be taken into consideration: 1) dishonest behaviour in the classroom is associated with dishonest behaviour in the clinical setting; 2) even the slightest occurrence of dishonest behaviour in the clinical setting can lead to fatal events.

**Conclusions:**

The gradual increase in BSc nursing students’ dishonest behaviour with their year of study raises several questions regarding the development of ethical and moral values in this population. This raises the need for early and continuous exposure of students to ethical content from the beginning of their studies and support from competent educators.

## Introduction

The rapid development of modern technology and the pervasive phenomenon of credentialism and academic inflation^1,2^ seem to contribute to a significant increase in unethical and academic dishonest behaviour among nursing students worldwide.^3–7^ Credentialism and educational inflation encompass a number of interrelated processes that include excessive formal demands and ‘*inflated*’ needs for high grades for the purpose of academic and professional success, increased chance of employment and career advancement. The mentioned competition policy performs additional pressure on students who strive to achieve excellent grades at all costs.^3–7^

Academic dishonesty in higher education is a widespread, insidious and global problem.^8^ In higher education, nursing programmes can have profound negative implications for the clinical setting. There is a serious risk that nursing students who behave dishonestly in the classroom will behave similarly also in the clinical setting, thus directly compromising patient safety and the quality of health care.^4,6,7,9^

While most studies in this area focus on nursing students’ academic dishonesty in the classroom, only a few relevant studies focus on nursing students’ academic dishonesty in the clinical setting.^7,9–14^ This study attempts to fill this gap by examining the profile of dishonest behaviour among nursing students and exploring the relationship between such behaviour in the classroom and in clinical practice.

## Background

Academic integrity is considered to be ‘*the prevalence of honesty in all academic matters*’, and any violation of academic integrity is considered academic dishonesty.^15^ Different theories attempt to explain the phenomenon of academic dishonesty from different perspectives. For example, Bandura’s *Social Learning Theory* provides a comprehensive theoretical framework for research in the context of nursing students.^14^ On the other hand, Kohlberg’s *Stages of Moral Development* and Watson’s *Theory of Human Caring*^16^ offer specific conceptual explanations for academic dishonesty. From a conceptual perspective, academic dishonesty is defined as the intentional deception concerning one’s own academic work or the work of others.^6,9,15^ This type of misconduct typically involves some form of deception or fraudulent activity and may include plagiarism, collusion, forging assessors’ signatures in the practice assessments or cheating during exams.^15^ Also, academic misconduct includes helping others to commit an academic offence.^5,6,15^ The operationalised aspect includes estimates of academic dishonesty through observation or self-assessment methods. This can be done through qualitative research approaches (e.g. interviews, observations) that help to understand the reasons and motivations for academic dishonesty, while quantitative (e.g. structured, validated questionnaires) allow the examination of incidence, attitudes and express them in numbers.^15,17^

DiPietro’s^18^ review of the literature addresses five basic theoretical frameworks on cheating and academic integrity: Deterrence Theory, Rational Choice Theory, Neutralization Theory, Planned Behaviour Theory and Situational Ethics. Most authors researching the phenomenon of academic dishonesty in nursing education describe nursing students’ opinions and attitudes towards it, and their studies are largely based on Sykes and Matze’s Neutralization Theory.^6,9,15^ This theoretical framework explains an individual’s dishonest behaviour using specific neutralisation techniques when individuals, especially adolescents, believe that their rationalisations of unfair practices are logical and fair, even if they do not conform to legal and social norms. Individuals thus employ neutralisation techniques to reduce feelings of guilt about their own dishonest behaviour.^15^

A significantly smaller number of studies examine the incidence of dishonest behaviour among nursing students, particularly in clinical settings. They are mainly based on Bandura’s Social Learning Theory.^7,13,14^ By considering the researched phenomenon of academic dishonesty and its cognitive and behavioural determinants (personality traits, opinions, attitudes, emotions, experiences and environmental influences), this study also has an underpinning in Bandura’s theory.

The analysis of student responses in this study provides a deeper insight into individual dishonest behaviours that students choose to apply more or less frequently in the classroom and clinical setting. In their responses, students acknowledge that in certain contexts they decide whether or not to perform certain dishonest behaviour and how often. The previously mentioned theoretical framework of Rational Choice provides the basis for understanding, interpreting and making meaning of student responses.^19^ This theoretical framework refers to academic dishonesty as the outcome of a person’s rational decision. The ultimate course of action is chosen after weighing the advantages and disadvantages of all possible alternatives. Therefore, the decision (not) to perform a particular dishonest behaviour is the result of weighing all possible consequences and benefits. The factors involved in the decision-making might include the effort involved in performing dishonest behaviour rather than performing actions aimed at improving the learning outcomes. Hypothetically, as well as empirically, nursing students have a considerably higher risk for more serious consequences if they engage in dishonest behaviour in real clinical environments.^4,6,7,9^

The study of academic dishonesty in nursing students has had a long tradition.^3–7^ As early as 1964, Bowers reported that 75% of the nursing students in his study had engaged in academic dishonesty.^10^ McCabe et al.^11^ also reported that nearly 58% of the 2000 participants had engaged in dishonest behaviour in the classroom. Furthermore, Theart and Smit^20^ noted that as many as 88% of nursing students confirmed that they had committed dishonest behaviour at least once. According to Nick and Llaguno,^3^ 80% of nursing students had performed dishonest behaviour in the classroom. Also, Park et al.^12^ reported that 76.8% of 655 nursing students had committed one or more forms of academic dishonesty in the classroom. Moreover, Oran et al.^21^ reported that 41.7% of health science students sometimes engaged in dishonest behaviour. Also, Anoopa et al.^22^ reported that nearly 84% of nursing students had witnessed academic dishonesty among their peers.

Some authors^4,6,10,14,15,23,24^ suggest that students’ academic dishonesty in the classroom is associated with academic dishonesty in clinical practice. Given the continued global increase in academic dishonesty in the classroom by nursing students, there is a legitimate risk and concern that healthcare facilities will become a potentially unsafe and risky environment for patients during clinical practice.^14,15^ According to Lovrić et al.,^6^ students who fail to maintain academic integrity during their studies will also fail to act with integrity in their future professional and personal relationships.

Previous similar quantitative studies on academic dishonesty in nursing students have been based on different research instruments developed for the purpose of each study and conducted in different sociocultural contexts. This makes a comparison of their results difficult or impossible. In many cases, the psychometric characteristics of these instruments (i.e. validity, reliability) were not reported. Therefore, this study attempts to fill this gap by using a validated instrument to investigate the situation of academic dishonesty in the Croatian context and compare its findings with those of similar studies in other countries. This will advance the understanding of the global phenomenon of academic dishonesty in nursing students.

## Research objectives

The objectives of this study were to determine, by student self-assessment:

1. The incidence of student dishonest behaviour in the classroom and clinical settings
2. The differences in the aforementioned incidence in the classroom and clinical setting considering the participants' level of study (BSc/MSc).
3. The relationship between the incidence of student dishonest behaviour in both settings.

## Methods

### Research design

This was a quantitative cross-sectional study using an online questionnaire for data collection.

### Instrument

In this study, we used the Croatian version of the *Nursing Student Perceptions of Dishonesty Scale* (CRO-NSPDS).^6^ The original instrument was developed in the USA by McClung and Schneider^17^ based on their expertise and a detailed analysis of the theories in the field as well as their findings from qualitative and quantitative studies.

Prior to the current study, all necessary steps for the translation, cross-cultural adaptation and psychometric evaluation of the Croatian version of the NSPD were performed to obtain a valid and reliable instrument for this and future studies.^6^ The instrument was tested on 733 Croatian nursing students, the results of psychometric analysis indicate: high reliability (the overall Cronbach α was in this study was 0.930, stability over time) and high level of content and construct validity.^6^

The instrument applied in this study comprised of two sections: the first section included questions for collecting demographic and other general data (e.g. gender, age, year of the study, BSc/MSc study, full/part time study, marriage, etc.), while the second section comprised 56 CRO-NSPDS items (dishonesty behaviours), representing nine subscales (Tables 2 and 3). Thus, the CRO-NSPDS measures the incidence of dishonest behaviour of nursing students in the classroom using six classroom subscales: *Cheating* (actions given or taken in an attempt to do well on the test or assignments without doing the actual work)*, Assistance* (to improve one’s work with the help of others)*, Cutting Corners* (actions taken to lessen the amount of work to be done)*, Not My Problem* (being aware of the academic dishonesty of others but not reporting it)*, Sabotage* (negatively impacting another’s work)*, Test File* (maintaining or using former tests or test question banks); and three clinical subscales: *Perjury* (creating or providing false or inaccurate information, to make up or lie)*, Non-Compliance* (failing to follow set guidelines, rules, or stated expectations)*, Stealing* (to take without permission or right).

Self-assessment of the incidence of 56 dishonest behaviour in the last semester was based on a 3-point scale (0=never, 1=once, 2=twice or more). Internal consistency (Cronbach’s alpha) of responses from the current study (Cheating: 0.886, Assistance: 0.788, Cutting corners: 0.791, Not my problem: 0.809, Sabotage: 0.911, Test file 0.813 Perjury: 0.942, Non-Compliance 0.912, Stealing: 0.721, overall Cronbach alpha: 0.933) was adequate, an exploratory factor analysis (varimax rotation method, factor loadings cut-off value: 0.4) confirmed the factor structure, which in accordance with the above mentioned study.^6^ All items loaded as expected to the corresponding factors, all factor loadings were above 0.5, the eigenvalues of the corresponding factors were >1.

### Participants

The participants in the study were BSc and MSc nursing students from the Higher Education Institution in Osijek, Croatia, EU, which was the first institution in this country to implement the graduate nursing study according to the EU Directives 2005/36/EC. This directive states that Bachelor of Science (BSc) nursing students in Croatia should complete 3 years of study with a minimum duration of 4600 h, of which 2300 h in clinical setting. MSc students, that is, the fourth and fifth years of study, complete a course with a minimum duration of 2100 h, of which 500 are in clinical practice. Classroom and clinical teaching and methods of formative and summative evaluation and assessment of students (i.e. knowledge, skills, autonomy and responsibility) are performed by considering elements and evaluation criteria, set by the institution. Students perform clinical training under the supervision of a clinical mentor according to the curriculum and a predefined clinical practice schedule. These are conducted in various nursing wards (e.g. surgery, otorhinolaryngology, neurology, internal medicine, orthopaedics, gynaecology, paediatrics, oncology, infectious disease clinic, psychiatry, nursing homes and health centres, etc.). Regular rotation of groups ensures that all students have the opportunity to clinically train in all departments.

The sample size calculation was based on the total number of nursing students studying at the surveyed higher education institution in the academic year 2020/2021 (*n = 546*), with an initial defined confidence interval value of 3%, a confidence level of 95% and an α-level of 0.05.^25^ According to the calculations of this study, the lowest required sample size was 361 participants. In addition, we assessed whether the recommended sample size met the requirements for sufficient statistical power of 0.8 for the statistical tests used (a priori).^26^ The post-hoc assessment also indicates sufficient statistical power. The inclusion criteria were: 1) BSc and MSc nursing students, 2) who in the academic year 2020/2021 from the surveyed institution and 3) participated voluntarily in the study. All 546 eligible nursing students were invited to participate, 19 students (3.48%) refused to participate), 81 (14.84%) students did not respond to the invitation.

Therefore, the final sample was homogeneous and consisted of 446 students (response rate: 81.68%), of whom 384 (86.1%) were female, and 62 (13.9%) were male (Table 1). Participants were 19 to 59 years old, and the average participant age (mean) was 27.4 years (SD = 8.1). The participants' detailed demographic data are presented in Table 1.

**Table 1.** Demographic characteristic of participants (*n* = 446).

Characteristic		*n* (%)
Gender	Male	62 (13.9)
	Women	384 (86.1)
Study year	BSc – 1st year	99 (22.2)
	BSc – 2nd year	101 (22.7)
	BSc – 3rd year	96 (21.5)
	MSc – 4th year	70 (15.7)
	MSc – 5th year	80 (17.9)
Part/full time study	Full time	247 (55.3)
	Part time	199 (44.6)
Marriage	Single	308 (69.1)
	Married	138 (30.9)
Have children	Yes	154 (34.5)
	No	292 (65.5)
Employed in healthcare sector	Yes	223 (50.0)
	No	223 (50.0)
Area of residence	Urban	311 (69.7)
	Rural	135 (30.3)

### Data collection

Data were collected using the Google Forms web survey tool between 1 August and 1 September 2020. An email with the link to the survey was sent to all potential participants. The introductory part of the email contained the purpose of the study and the guidelines for completing the questionnaire.

### Data analysis

Data analysis was conducted using SPSS 17.0, *Sample size calculator*^25^ and *GPower* 3.1.9.7^26^. Descriptive statistics for nominal variables were expressed as frequencies and percentages, while mean (M) and standard deviation (SD) were used for numerical variables. Due to substantial departures from the normal distribution, the Mann–Whitney *U* test was used to compare the differences in responses between BSc and MSc students, and the Kruskal–Wallis test was used to compare the differences in responses between participants’ years of study. Spearman’s correlation coefficient (r_s_) was used to calculate the association between different subscales. As mentioned earlier, the internal consistency of the responses was measured using Cronbach’s alpha and factor analysis was used to examine the factor structure of the data. The statistical significance level was 0.05, and the statistical power was 0.8.

### Ethical considerations

The study was approved by the committee of the institution where the study was conducted (institutional review board approval number: 2158-61-07-18-14). Participation in the study was voluntary and students had the right to withdraw from the study without consequence. Before responding to the questionnaire, participants were informed about the purpose of the study, detailed ethical aspects and expected research outcomes. In addition, the institutional review board consent form was available as a link. Informed consent was implied by answering the appropriate question and voluntarily completing the questionnaire. We would like to acknowledge that an incentive was given to improve the response rate. Students in the year with the highest percentage of responses were exempt from paying the student fee for a scientific conference organised by the institution where the study was conducted. To improve the quality of participants’ responses and to relieve them of the fear or shame of reporting past dishonest behaviours, the study was conducted after the end of courses, clinical practice and exams. The online questionnaires were completed outside of the institution’s facilities without faculty supervision; students were free to choose the time and place to complete the questionnaire.

## Results

The overall mean value of the incidence of dishonest behaviour among all participants (*n = 446*) according to the NSPDS scale (response range 0–2) was 0.33 (*SD = 0.25*): 0.54 (*SD = 0.31*) in the classroom and 0.12 (*SD = 0.28*) in the clinical setting. Of the 446 participants, in the last semester 13 (2.9%) had committed an act of dishonest behaviour only once in the classroom, while 82 (18.4%) had done so in the clinical setting. In addition, 407 (91.3%) had engaged in dishonest behaviour two or more times in the classroom, while 145 (32.5%) had done so in the clinical setting.

### Incidence of nursing students’ dishonest behaviour in the classroom setting

A detailed analysis of the five most frequent dishonest behaviours (rank 1-5) in the classroom shows no significant differences between the responses of BSc and MSc students (please, refer to column rank in Table 2). In fact, items 16 (*A ‘S’ answers questions from a classmate about how to complete the assignment*) and 14 (*A ‘S’ asks a classmate to explain the homework instructions*) of the *Assistance* subscale had the highest incidence in both groups, two were from the *Cheating* subscale: item 4 (*During the exam a ‘S’ looks at a classmate’s test to compare answers*) and 8 (*During an exam a ‘S’ allows a classmate to view her answers*). Furthermore, the next five most frequent dishonest behaviours (rank 6-10) were also represented by the items from the *Assistance, Cheating* and *Not My Problem* subscales.

**Table 2.** Incidence of nursing students’ dishonest behaviour in the classroom (*n* = 446).

Subscales Items	Undergraduate students (*n* = 296)					Graduate students (*n* = 150)				
	Percentage (%)			Mean	Rank	Percentage (%)			Mean	Rank
	Never	Once	Twice or more			Never	Once	Twice or more		
Cheating										
1. During the exam a ‘S’ receives answers via text message	86.5	7.4	6.1	0.20	19	84.0	4.7	11.3	0.27	19
2. Texting answers to another ‘S’ during an exam	88.2	5.4	6.4	0.18	21	82.0	6.7	11.3	0.29	18
3. During the exam a ‘S’ uses a cheat sheet.	46.6	25.7	27.7	0.81	11	34.7	26.0	39.3	1.05	8
4. During the exam a ‘S’ looks at a classmate’s test to compare answers	18.2	24.7	57.1	1.39	4	19.3	28.7	52.0	1.33	4
5. A ‘S’ has someone else take her exam	97.0	1.4	1.7	0.05	28	96.0	2.7	1.3	0.05	29
6. A ‘S’ takes an exam for another ‘S’	98.6	0.3	1.0	0.02	32	94.7	4.0	1.3	0.07	27
7. ‘S’ shares answers with a classmate through a predetermined communication system such as hand signals	46.6	17.9	35.5	0.89	8	57.3	18.7	24.0	0.67	11
8. During an exam a ‘S’ allows a classmate to view her answers	15.2	13.5	71.3	1.56	2	9.3	26.7	64.0	1.55	2
9. A ‘S’ submits a paper he purchased	91.6	4.1	4.4	0.13	24	92.0	6.0	2.0	0.10	26
10. A ‘S’ writes a paper for another ‘S’	87.5	7.1	5.4	0.18	20	78.0	12.0	10.0	0.32	17
11. A ‘S’ submits the work of another ‘S’ as his own	93.6	3.4	3.0	0.09	26	90.7	6.0	3.3	0.13	23
12. A ‘S’ sells a lab report to another ‘S’	98.0	0.3	1.7	0.04	30	96.0	2.0	2.0	0.06	28
Assistance										
13. A ‘S’ had a friend edit a paper for grammatical and spelling errors	40.5	32.8	26.7	0.86	9	36.0	24.7	39.3	1.03	9
14. A ‘S’ asks a classmate to explain the homework instructions	12.5	29.1	58.4	1.46	3	16.0	29.3	54.7	1.39	3
15. A ‘S’ edits a friend’s paper for writing style and organisation	37.5	31.4	31.1	0.94	7	28.0	20.0	52.0	1.24	7
16. A ‘S’ answers questions from a classmate about how to complete the assignment	9.5	24.0	66.6	1.57	1	10.0	24.0	66.0	1.56	1
17. A ‘S’ edits a friend’s paper for grammatical and spelling errors	45.3	26.0	28.7	0.83	10	23.3	21.3	55.3	1.32	5
Cutting corners										
18. A ‘S’ assigned to read a novel watches the film version instead	75.3	12.8	11.8	0.36	15	71.3	12.0	16.7	0.45	14
19. A ‘S’ reads the Croatian version of a novel that was assigned to be read in English	75.0	15.2	9.8	0.35	16	66.0	14.7	19.3	0.53	13
20. When a novel is assigned, a ‘S’ reads a summary instead	54.1	19.6	26.4	0.72	12	58.0	21.3	20.7	0.63	12
21. A ‘S’ submits a paper with larger font size and punctuation than allowed	88.5	8.8	2.7	0.14	22	89.3	7.3	3.3	0.14	22
22. A ‘S’ submits a paper with larger margins than allowed	90.2	6.8	3.0	0.13	23	92.7	4.0	3.3	0.11	24
Not my problem										
23. A ‘S’ witnesses cheating during an exam and does not report it	38.2	11.5	50.3	1.12	5	29.3	15.3	55.3	1.26	6
24. A “S” witnesses cheating during a lab practical and does not report it	85.1	4.4	10.5	0.25	18	90.0	4.7	5.3	0.15	21
25. A ‘S’ is aware of plagiarism and does not report it	75.3	9.5	15.2	0.40	14	82.0	8.7	9.3	0.27	20
26. A ‘S’ is aware of a previous test being passed around and does not report it	45.9	14.2	39.9	0.94	6	39.3	18.7	42.0	1.03	10
Sabotage										
27. A ‘S’ destroys a classmate’s lab experiment	95.6	2.7	1.7	0.06	27	96.7	2.0	1.3	0.05	30
28. ‘S destroys library reserve material	98.0	1.4	0.7	0.03	31	97.3	2.0	0.7	0.03	31
29. So another ‘S’ cannot complete the assignment, a ‘S’ intentionally breaks a DVD.	96.6	2.7	0.7	0.04	29	98.7	1.3	0	0.01	32
30. A ‘S’ does not participate in group work	81.1	9.8	9.1	0.28	17	79.3	6.0	14.7	0.35	16
Test file										
31. A ‘S’ studies test bank questions	60.5	13.9	25.7	0.65	13	72.0	15.3	12.7	0.41	15
32. A ‘S’ maintains a test file	92.6	3.0	4.4	0.12	25	93.3	2.7	4.0	0.11	25

### Incidence of nursing students’ dishonest behaviour in clinical settings

Similarly, as in classroom settings, there were no relevant differences between the responses of BSc and MSc students related to the five most commonly (rank 1–5) performed dishonest behaviours in clinical settings (please, refer to column rank in Table 3). For both of them, the item 55 (*A ‘S’ uses a band aid from the supply room to cover a cut*) of the *Stealing* subscale was ranked first. In addition, three items from the *Perjury* subscale were also in this top five list for both BSc and MSc students: item 39 (*A ‘S’ makes up vital signs and documents them*) item 40 (*Instead of completing the patient assessment, a ‘S’ documents the nurse’s assessment data as her own*) item 42 (*A ‘S’ makes up an entire patient assessment and documents it*). This list also included item 37 (*A ‘S’ documents that lung sounds are clear, when the assessment was never completed*) from the *Perjury* subscale for BSc students, and item 48 (*A ‘S’ does not wash his hands between patients*) from the *Non-Compliance* subscale for MSc students.

**Table 3.** Incidence of nursing students’ dishonest behaviour in the clinical settings (*n* = 446).

Subscales Items	BSc students (*n* = 296)					MSc students (*n* = 150)				
	Percentage (%)			Mean	Rank	Percentage (%)			Mean	Rank
	Never	Once	Twice or more			Never	Once	Twice or more		
Perjury										
33. When reporting off to the nurse, a ‘S’ says bathing was completed when it was not	97.0	1.0	2.0	0.05	14	92.0	4.7	3.3	0.11	16
34. A ‘S’ documents ROM was completed and it was not	96.3	2.0	1.7	0.05	15	91.3	3.3	5.3	0.14	10
35. A ‘S’ makes up a pain score rather than asking the patient	89.9	5.1	5.1	0.15	4	90.0	2.0	8.0	0.18	7
36. A ‘S’ documents that the patient was turned every 2 h when it was not done	92.9	3.4	3.7	0.11	7	88.7	3.3	8.0	0.19	4
37. A ‘S’ documents that lung sounds are clear, when the assessment was never completed	89.5	7.4	3.0	0.14	5	90.0	2.0	8.0	0.18	8
38. When reporting off to the nurse, a ‘S’ says the patient refused to eat, when the ‘S’ did not even ask the patient	95.9	2.7	1.4	0.05	16	94.7	2.0	3.3	0.09	17
39. A ‘S’ makes up vital signs and documents them	86.1	8.1	5.7	0.20	3	86.7	7.3	6.0	0.19	5
40. Instead of completing the patient assessment, a ‘S’ documents the nurse’s assessment data as her own	84.5	9.8	5.7	0.21	2	84.7	7.3	8.0	0.23	3
41. A ‘S’ documents that the nurse was notified when the nurse was not notified	94.6	3.0	2.4	0.08	9	90.0	6.0	4.0	0.14	11
42. A ‘S’ makes up an entire patient assessment and documents it	93.6	4.4	2.0	0.08	10	88.7	5.3	6.0	0.17	9
43. A ‘S’ documents hourly rounding was completed when it was not done	92.6	4.7	2.7	0.10	8	87.3	6.0	6.7	0.19	6
44. A ‘S’ gives a patient inaccurate information about his status	97.3	1.7	1.0	0.04	19	92.0	3.3	4.7	0.13	13
45. When asked to witness a medication waste, the ‘S’ signs off but did not watch to ensure the drug was wasted	98.0	0.7	1.4	0.03	20	94.0	2.7	3.3	0.09	18
Non-compliance										
46. After dropping a medication on the floor, as ‘S’ picks it up and administers it to the patient	93.6	4.4	2.0	0.08	11	92.0	3.3	4.7	0.13	14
47. A ‘S’ fails to report a patient fall	98.0	1.0	1.0	0.03	21	95.3	2.0	2.7	0.07	23
48. A ‘S’ does not wash his hands between patients	91.9	4.1	4.1	0.12	6	81.3	8.7	10.0	0.29	2
49. A ‘S’ accepts a cash gift from a patient	95.3	3.7	1.0	0.06	13	94.0	4.0	2.0	0.08	21
50. A ‘S’ breaks sterility and does not reestablish it	94.9	3.4	1.7	0.07	12	90.0	6.0	4.0	0.14	12
51. A ‘S’ discovers a medication error and does not report it	98.0	0.7	1.4	0.03	22	94.0	4.0	2.0	0.08	22
52. A ‘S’ does not follow a physician’s order	96.3	2.4	1.4	0.05	17	91.3	4.7	4.0	0.13	15
53. A ‘S’ makes a medication error and does not report it to the instructor	98.0	0.7	1.4	0.03	23	96.7	0.7	2.7	0.06	24
Stealing											
54. A ‘S’ eats graham crackers from the patient pantry	98.6	0.7	0.7	0.02	24	93.3	4.7	2.0	0.09	19
55. A ‘S’ uses a band aid from the supply room to cover a cut	63.2	24.3	12.5	0.49	1	58.0	26.0	16.0	0.58	1
56. A ‘S’ takes and uses TEDs/surgical stockings from the supply room for personal use	97.0	0.7	2.4	0.05	18	94.0	3.3	2.7	0.09	20

### Differences in the incidence of dishonest behaviour between classroom and clinical settings

**Figure 1.**
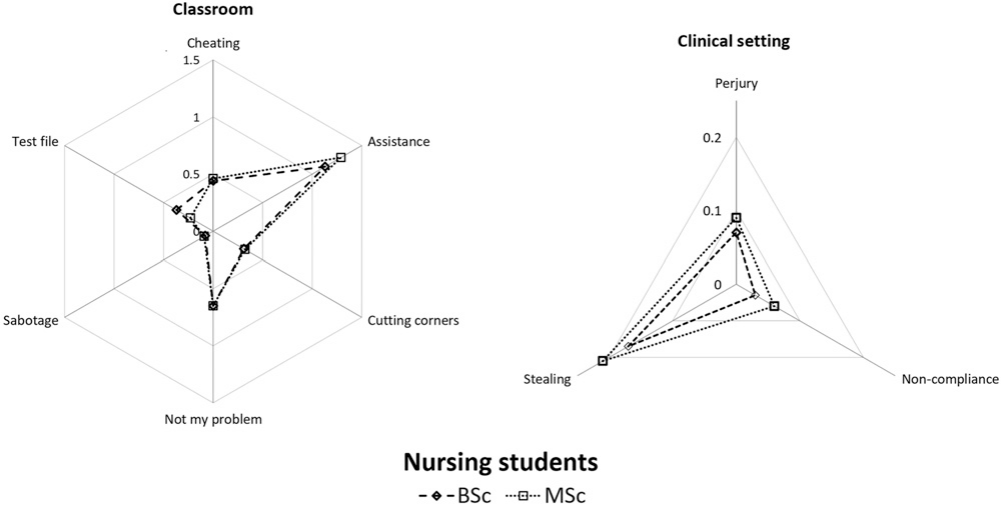
The profile of study participants’ dishonest behaviour.

For a clearer overview of the profile of our participants’ dishonest behaviour, the mean scores of the incidence of dishonest behaviour related to different subscales are presented in Table 4 and graphically displayed in the radar chart in Figure 1 (Section: Classroom), where each axis represents the mean incidence of dishonest behaviour for each subscale for BSc and MSc students, respectively. Similarly, the mean scores of dishonest behaviour incidence in the clinical setting are presented in the same figure in the corresponding section.

**Table 4.** Comparison of dishonest behaviour incidence between BSc and MSc students (*n* = 446).

Group Subscale	Study programme		Mann–Whitney *U* test	
	BSc Mean	MSc Mean	U	*p*
Classroom	0.51	0.53	19,399.5	.453
Cheating	0.44	0.46	20,156.5	.902
Assistance	1.13	1.29	17,051.0	.007*
Cutting corners	0.31	0.32	19,921.5	.738
Not my problem	0.65	0.65	19,850.0	.701
Sabotage	0.08	0.09	20,221.0	.921
Test file	0.37	0.23	17,489.0	.006*
Clinal settings	0.07	0.09	19,700.0	.590
Perjury	0.07	0.09	19,891.5	.635
Non-compliance	0.03	0.06	18,746.5	.037*
Stealing	0.17	0.21	19,004.5	.211

*p < .05.

Significant differences were found in the incidence of dishonest behaviour between the years of study (*p = 0.008*). The lowest mean (0.25) was reported by Year 1 students, 0.34 by Year 2 students and 0.35 by Year 3 students (BSc study programme); the highest mean incidence was reported by Year 4 students, that is, 0.43, and 0.30 by Year 5 students (MSc study programme).

The results of the Mann–Whitney *U* test showed no statistically significant differences between BSc and MSc students in the overall mean scores for classroom (*M*_*BSc*_ *= 0.51, M*_*MSc*_ *= 0.53, U =* 19,399.5*, p* = 0.453) and clinical settings (*M*_*BSc*_ *= 0.07, M*_*MSc*_ *= 0.09, U = 19,700.0, p = .590*) (Table 4). However, further analysis of the classroom subscales reveals significant differences in responses between BSc and MSc students for the *Assistance* subscale *(p* = .007*)*, where the mean incidence of dishonest behaviour was higher for MSc (*M*_*MSc*_ *= 1.29, M*_*BSc*_ *= 1.13*) participants, in contrast to the *Test File* subscale *(p* = .006*),* where it was higher for BSc participants (*M*_*BSc*_ *= 0.37, M*_*MSc*_ *= 0.23*). For the clinical setting subscales, the results of the Mann–Whitney *U* test show a significant difference between the responses of undergraduate and graduate students for the *Non-Compliance* subscale *(p = .006)*, where the mean incidence of dishonest behaviour was higher in MSc (*M*_*MSc*_ *= 0.06*) than in BSc (*M*_*BSc*_ *= 0.03*) students (Table 4).

### Relationship between dishonest behaviour in the classroom and in the clinical setting

Our results show a statistically significant, positive, moderate correlation between the mean of all subscales for the classroom setting and all subscales for the clinical setting (*r*_*s*_ *= 0.393; N = 446; p < .001*). A detailed analysis of the correlations between each *Classroom* subscale and each *Clinical Setting* subscale (Table 5) revealed a positive, moderate, positive correlation between the *Sabotage* and *Perjury* subscales (*r*_*s*_ *= 0.418; N = 446; P <.001*). Only one correlation, namely, that between *Not My Problem* and *Non-Compliance*, proved to be non-significant, while others were statistically significant, positive, but weak (*r*_*s*_ *< 0.3*). Most of these correlations yielded adequate statistical power (above 0.8), with the exception of *Cutting Corners* and *Non-Compliance* as well as *Sabotage* and *Stealing*, which scored slightly below the minimum value of 0.8. The correlations between all classroom subscales and *Perjury* ranged from 0.196 to 0.418, which is slightly higher than the correlations between all classroom subscales and *Non-Compliance* [0.091, 0.261] and also all classroom subscales and *Stealing* [0.123, 0.204].

**Table 5.** Spearman correlation coefficients between classroom and in clinical settings dishonest behaviour subscales (*n* = 446).

	Clinical settings subscales					
Classroom subscales	Perjury		Non-compliance		Stealing	
	r_s_	1-β	r_s_	1-β	r_s_	1-β
Cheating	0.253**	0.999	0.212**	0.995	0.204**	0.992
Assistance	0.196^**^	0.987	0.186^**^	0.978	0.186^**^	0.977
Cutting corners	0.217^**^	0.996	0.128^**^	0.774	0.133^**^	0.805
Not my problem	0.250^**^	1.000	0.091	NC	0.168^**^	0.947
Sabotage	0.418^**^	1.000	0.261^**^	1.000	0.123^*^	0.741
Test file	0.280^**^	1.000	0.205^**^	0.992	0.196^**^	0.987

r_s_ – Spearman correlation coefficient; 1-β: statistical power; * *p* < .05; ** *p* < .01; NC – Not Calculated.

## Discussion

The results of this study show a relatively low mean dishonest behaviour incidence score of 0.33 (response range 0–2): 0.54 in the classroom, and 0.12 in the clinical setting. At first glance, this result can be interpreted as satisfactory, but considering the possible consequences of dishonest behaviour in the clinical setting, even the lowest incidence must be taken seriously.^6,9^

The profile of participants’ incidence of dishonest behaviour in the classroom by subscale (Figure 1) shows the highest mean frequency for the *Assistance* subscale (1.19) and the lowest for *Sabotage* (0.11), while in the clinical setting the highest mean incidence is seen in the *Stealing* subscale (0.21) and the lowest in the *Non-Compliance* subscale (0.08). These results are not surprising, as students most often perceive the *Assistance* (*Classroom*) and *Stealing* (*Clinical Setting*) subscales as least dishonest, while they perceive the *Sabotage* (*Classroom*) and the *Non-Compliance* subscales (*Clinical Setting*) as most dishonest.^15^ Comparable results were reported by McClung^15^ where the subscale *Assistance* was perceived as least dishonest by the students. The behaviours of this subscale were performed by 85% of the students. In contrast, the subscale *Sabotage* was perceived as extremely dishonest, with only 1% of students performing the behaviours from this subscale. Also, the behaviours from the *Non-compliance* subscale were only performed by 8% of the students. Thus, students clearly behaved in accordance with their attitudes, which supports the psychological thesis that students' behavioural patterns depend significantly on their perceptions, beliefs and values.^14^ Participants' responses can also be interpreted from the perspective of Rational Choice Theory,^19^ previously described in the Background section. It is evident that students were more likely to perform dishonest behaviours with a lower risk of serious consequences than those with serious consequences that endanger other people’s lives/property and may have irreparable consequences for them.

### Incidence of dishonest behaviour in the classroom

The majority, or 91.3%, of participants have committed acts of dishonest behaviour in the classroom on two or more occasions, with a mean value of 0.54. In a study by McClung and Gabeersonn,^9^ as many as 96% of students reported committing at least one dishonest act, and 60% reported committing five or more. In the study of Rafati,^7^ 89.1% of nursing students reported engaging in at least one form of dishonest behaviour in the previous semester. A high incidence of dishonest behaviour, that is, 88%, is also reported by Theart and Smit,^20^ in which 12% of the participants never engaged in such behaviour. This percentage was much lower in our study (5.8%). Kiekkas et al.^27^ also reported that 51% of nursing students engaged in dishonest behaviour, and according to Bloomfield et al.,^28^ 44.1% of students reported committing with at least one type of dishonest behaviour. Compared to our study, researchers from different continents and cultures report lower incidence rates of dishonest behaviour: For example, McCabe^11^ (58%), Park et al.^13^ (76.8%), and Oran^21^ (41.7%).

Detailed analysis of the incidence of dishonest behaviour in classroom (Table 2) shows that BSc and MSc students most often asked or helped their fellow students (*Assistance* subscale), followed by cheating on written examinations (*Cheating* subscale) and witnessing and not reporting dishonest acts committed by their fellow students (*Not My Problem* subscale). The high incidence of dishonest behaviour corresponding to the *Assistance* subscale is not surprising, as according to the authors of the NSPDS,^15^ the corresponding items cannot be considered as acts of dishonest behaviour. More than 80% of participants in both the BSc and MSc groups frequently looked at a fellow student’s test to compare their answers or allowed their fellow student to see their answers (*Cheating* subscale). This is consistent with other studies.^3,4,10,12^ In contrast, some studies report that dishonest behaviour associated with *Cheating* is rare, with various forms of plagiarism predominating.^11,20,27^ More than 60% of all participants in our study reported that they frequently witnessed dishonest behaviour in their fellow students and did not report it to anyone, which is consistent with other similar studies.^11,20,27^ Students often perceive cheating as unfair, but are reluctant to report their fellow students in order to maintain friendly relationships and avoid the potential negative influence of peer pressure.^20^

### Incidence of dishonest behaviour in the clinical setting

Although the mean incidence of dishonest behaviour in the clinical setting (0.12) may seem insignificant, it cannot be ignored as more than half (51.0%) of all participants reported having engaged in dishonest behaviour on one or more occasions. Many authors warn of a high incidence of dishonest behaviour worldwide.^3–7,9–11,13,14^

In the clinical setting, the form of dishonest behaviour with the highest incidence was the unauthorised use of a patch/bandage, property of the medical facility and intended for use on patients to protect their own injury (*Stealing*) (Table 3) and was reported by more than one-third of all participants (38.6%). This is problematic because these materials are usually available to staff for such cases (e.g. as first aid supplies) and are strictly separated from patient supplies. Such behaviours are perceived by participants as less dishonest than others that pose a direct threat to patient safety (e.g. therapeutic errors).^6^ Unfortunately, according to McClung and Schneider,^15^ students also witnessed dishonest behaviour from the *Stealing* and *Non-Compliance* subscales performed by health professionals in clinical practice. Therefore, students may begin to believe that such behaviour is socially acceptable, which leads to it becoming common.

The most commonly reported dishonest behaviour was providing false or inaccurate information about the patient’s vital signs, breathing and pain (*Perjury* subscale). In addition, instead of completing the nursing assessment form with actual patient data, students reported copying or slightly modifying the values previously measured by others. Many authors rank such behaviour as one of the most dishonest acts in clinical practice.^10,29–31^ In our study, 15% of participants reported engaging in dishonest behaviour from this subscale, which is consistent with another study.^13^ Park et al.^13^ found that 39% of participants in their study reported not taking or recalling vital signs accurately. The high incidence of perjurious behaviour in the clinical setting is both surprising and concerning as students generally perceive the acts of dishonest behaviour included in the *Perjury* subscale as particularly unfair.^15^ From the perspective of causality, perjury is more of an ‘individual/private’ behaviour, unlike the dishonest acts included in the *Stealing* and *Non-Compliance* subscales, which can be observed by others.^15^ Furthermore, reporting and documenting false patient data often exposes students to uncomfortable feelings of guilt which require the use of self-justification and neutralisation techniques, and students appear to use these in practice much more frequently than is usually acknowledged.^9^ According to Sykes and Matze’s Neutralization Theory, students in such situations may deny false information and claim their truth. Students may also deny their own responsibility for dishonest behaviour and attribute the responsibility to other students or members of the healthcare team. In addition, students can appeal for a higher level of credibility with them while persistently denying their own guilt.^9^

In the clinical setting, MSc students' dishonest behaviour of the highest incidence was avoiding hand washing between treating individual patients (*Non-Compliance* subscale), which is surprisingly contrary to professional norms and principles. This behaviour was performed by almost 15% of the participants in our study. The fact that potential future nurses knowingly committed an act which posed a threat to patient safety is unacceptable. This form of dishonest behaviour is largely determined by individual factors (knowledge, attitude, experience, responsibility, habits, etc.), but unfortunately students may observe such behaviour in healthcare staff in clinical practice.^9,32^ According to a study conducted in the UK, 76.4% of nursing students witnessed healthcare workers failing to wash their hands between contacts with different patients.^32^ Such poor examples in clinical practice not only put patients at risk, but also significantly compromise the quality of clinical practice for students, as experiential learning has a critical impact on building students' future competencies.^33^

In the current study, 26 (5.8%) participants exhibited dishonest behaviour related to the application of therapy and 13 (2.9%) failed to report a patient fall, which is comparable to other similar studies.^13,34–37^ Stevanin^35^ reported that nursing students witnessed or reported a mean of 3.8 patient safety incidents in 1000 days of clinical practice in the hospital, and most of these incidents were related to the application of therapy. In addition, according to Reid-Searl et al.,^34^ approximately one-third of students were involved in activities which resulted in a therapeutic error. In addition, Park^13^ reported that 14.5% of students failed to report patient-related incidents or errors on one or more occasions. It is certain that nursing students contribute to the quality of healthcare and patient safety, which is why the results of this and other studies should be taken seriously. In the European Union, healthcare errors and incidents are recorded in 8–12% of hospital patients, and in the USA, 7000-9000 patients die due to medical errors.^38^ In this study, 23 (5.2%) participants reported accepting a monetary gift from a patient, which is considered an act of unethical and illicit behaviour. Students seem to observe this and similar behaviour in clinical practice where it is quite common for patients to give gifts to medical staff.^39^ Globally, there are quite different and conflicting views and regulations on accepting gifts from patients. Studies in this area are rare and have been conducted on small samples.^39^

### Differences in the incidence of dishonest behaviour between BSc and MSc students

The results of the current study show no statistically significant difference in dishonest behaviour between BSc and MSc students (Table 4). However, according to the NSPDS subscales, BSc students were significantly more likely to perform the acts of dishonest behaviour from the *Test-File* subscale (*Classroom*). This can be explained by the fact that there are more written examinations in BSc courses, whereas MSc courses are based on other forms of knowledge assessment (e.g. seminars, portfolio, project assignments, etc.).

In contrast, MSc students showed a significantly higher incidence of behaviour from the *Assistance* (classroom) subscale, which in principle cannot be considered as dishonest, but rather as academic support for their peers, as described earlier.^15^ This result is actually not surprising, as the MSc students in our study pursue a course of study which requires extensive student interaction for completing course activities (e.g. teamwork, group assignments and projects).

Unexpectedly, the mean incidence of dishonest behaviour seems to gradually increase over the years of BSc study: in Year 1, the mean incidence was lowest at 0.25, while in Year 4 it was 0.43. This result is consistent with Rafati et al.^7^ In addition, MSc students more frequently adopted the dishonest behaviour from the *Non-Compliance* (*Clinical Setting*) subscale. This is inconsistent with most previous studies, which suggest that younger students, especially Year 1 and Year 2 students, are more likely to engage in dishonest behaviour than older students.^4,20,40^ Students are expected to adopt and reinforce ethical and moral values during their studies.

### Relationship between dishonest behaviour in the classroom and dishonest behaviour in clinical settings

The results of our study show statistically significant correlations between the mean values of all *Classroom* and *Clinical Setting* subscales, with the exception of the *Not My Problem* and *Non-Compliance* subscales (Table 5). These correlations are consistent with the findings of previous studies.^4,10,12–14,23,24^ Thus, there is always a risk that unethical behaviour will be ‘transferred’ from the classroom to the clinical setting.^4,6,10^

To date, no clear explanations for dishonest behaviour have been suggested in the literature. In fact, the causality of dishonest behaviour seems to be multidimensional: For example, unclear definitions of dishonest behaviour, contextual factors, personality traits, peer behaviour, cultural beliefs and values, socio-demographic conditions, inconsistent implementation measures to enhance ethical awareness, inadequate supervision of teachers and mentors in clinical settings.^6,10,12–14,23,24^ It is therefore not surprising that different studies report different and even contradictory findings regarding dishonest behaviour in the classroom and in the clinical setting. Given these facts, there is a clear need for universities and healthcare institutions to continually invest in the development of policies aimed at improving students’ honesty and ethical attitudes, and conducting ongoing assessments of students' attitudes and behaviours.

The analysis of correlations between the *Classroom* and *Clinical Setting* subscales (Table 5) revealed a statistically significant, moderate, positive correlation between the *Sabotage* (*Classroom*) subscale and *Perjury* (*Clinical Setting*) subscale*.* This is interesting given that NSPDS authors McClung and Schneider^15^ report conceptual parallels and similarities in the behaviour related to *Perjury* (*Clinical Setting*) and *Cheating* (*Classroom*). The mentioned parallels were identified through an assessment of students’ perceptions regarding these behaviours. On the other hand, the significant correlation found between the *Sabotage* (*Classroom*) and *Perjury* (*Clinical Setting*) subscales is based on the incidence reported by students, that is, self-reported dishonest behaviour. We can assume that this correlation is due to the fact that acts of dishonest behaviour in the *Sabotage* (*Classroom*) and *Perjury* (*Clinical Setting*) subscales are predetermined by private factors as defined by McClung and Schneider,^15^ whereas dishonest behaviour of other subscales can be attributed to having witnessed such behaviour in others.

Furthermore, the non-significant correlation found between the *Not My Problem* (*Classroom*) and *Non-compliance* (*Clinical Setting*) subscales is also interesting as it provides an interesting insight into witnessing dishonest behaviour. Accordingly, we can assume that not reporting dishonest behaviour of other students is not necessarily related to the individual non-compliance in the clinical setting. Another interesting finding worth considering is the positive correlation of the *Perjury* (*Clinical Setting*) subscale with all of the *Classroom* subscales. Indeed, it seems to suggest that students who engage in dishonest behaviour in the classroom are more likely to also engage in acts of dishonest behaviour included in the *Perjury* (*Clinical Setting*) subscale (and vice versa). In extreme cases, this may seriously compromise patient safety and quality of care.

### Study limitations

There are several limitations to this study that should be considered. First, the data in this study were collected using a questionnaire. Even though respondents were asked to provide sincere responses and despite the fact that the survey was anonymous, they may not have provided entirely truthful responses. Second, the results collected represent self-reported behaviour. For this reason, additional data collection techniques should be used in the future to obtain more objective data about subjects’ actual behaviour. However, this raises several issues, as such observation of students conducted with their consent may lead to bias in their actual behaviour. It therefore remains a challenge to choose the research methods and data collection procedures that can overcome these problems. Third, students' responses only referred to dishonest behaviours, so positive examples of dishonest behaviours were not included, limiting this study to the ‘*dark side of the story*’. Furthermore, this study does not consider the reasons for their decisions, which limits our findings to presenting the current situation without providing an explanation. To fill this gap, a sequel of this study is planned to identify the predictors and causes of the academic dishonesty.

### Strategies for improving the ethical qualities and moral resilience of nursing students

To develop ethical qualities and moral resilience, nursing students should be continuously exposed to ethical content from the beginning of their studies. This can be done through the promotion of honour codes and honesty policies, the design and delivery of various courses, university education modules and lifelong learning programmes. Nursing educators must seize every opportunity to promote and enhance the ethical integrity of nursing students, for example, by promoting ethical sensitivity when ethical issues arise in the classroom or clinical settings.^15,29,41,42^ This should be done through the support of competent educators, who respect and care about these values.^43–45^ Nursing students should recognise in them a role model for whom dishonest behaviour is not an option.^6^

In addition, institutions should provide appropriate support to students, including mentoring, support groups and various events (e.g. conferences, workshops and discussions) to proactively, rather than reactively, expose students to the potential consequences of dishonest behaviour.^15^ Such discussions can change the way students think about their behaviour and have far-reaching effects on all areas of their lives.

## Conclusion

Our study confirms the presence of academic dishonesty in nursing students also in the Croatian context. The most worrying finding is the gradual increase in dishonest behaviour among BSc nursing students over their years of study, which raises several questions regarding the development of ethical and moral values in this population. There are several approaches to improve this situation: For example, reducing student workload, imposing strict punishments for such behaviour and supervision. Students should be aware of the consequences of dishonest behaviour, and in particular of the fact that, even if committed in the classroom, according to our findings, it can lead to dishonest behaviour in the clinical setting. Before this, however, further studies are needed to determine the causative factors of dishonest behaviour. Last but not least, all nursing educators should do their best to ‘cure’ this global problem by providing students with a valid role model and supporting them in their ethical and moral development. We suggest that we speak openly about the potential consequences of such behaviour on the integrity and reputation of the nursing profession. Finally, and importantly, our study introduces a new approach to profiling dishonest behaviour in a population using a radar chart. This representation facilitates the identification of problematic dishonest behaviours and assists management and teachers in their improvement initiatives.
